# Supplementary material for: Assessment of Different Circulating Tumor Cell Platforms for Uveal Melanoma: Potential Impact for Future Routine Clinical Practice
Source: Int J Mol Sci. 2023 Jul 4;24(13):11075. doi: 10.3390/ijms241311075 (PMC10342234; doi:10.3390/ijms241311075)
Supplement: Supplementary file 1 [file ijms-24-11075-s001.zip › ijms-2459151-supplementary.pdf]

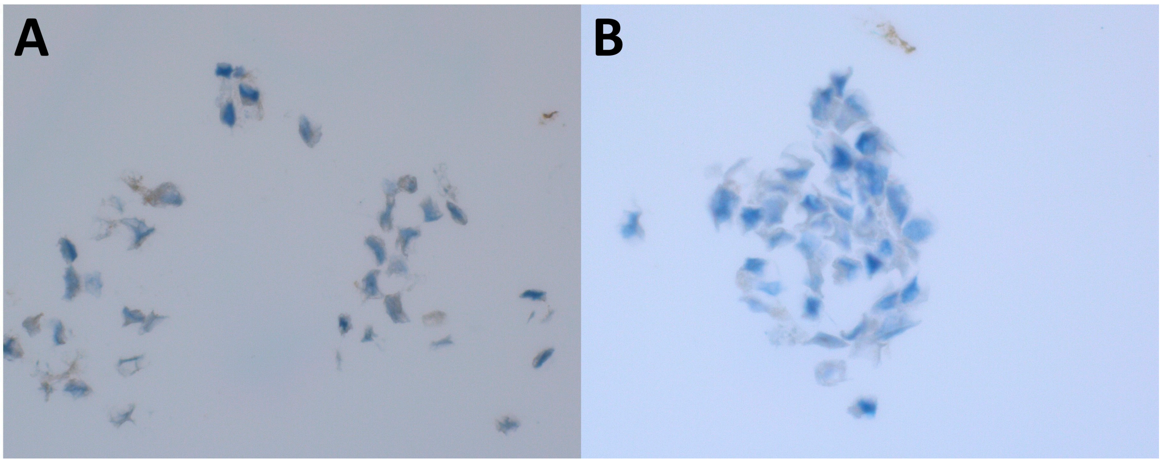

Supplementary Figure S1. expression in melanoma cell lines: A: M229 cell line. B: OMM 2.3 cell line. (immuno-peroxidase; clone MUC18/1130, ab233923, Abcam Plc, UK; original magnification x400).
